# Impact of COVID-19 on cutaneous squamous cell carcinoma severity in a tertiary referral center in Israel: Lessons for future pandemics

**DOI:** 10.1016/j.jdin.2025.09.003

**Published:** 2025-09-29

**Authors:** Deborah Korn, Sivan Sheffer-Levi, Maayan Eitan-Wexler, Stephanie Ben-Shushan, Mordechai Avner, Nir Hirshhorn, Meni Bracha, Alexander Mali, Roni Shereberk-Hasidim, Sharon Merims, Aron Popovtzer, Vered Molho-Pessach, Michal Lotem, Jonathan E. Cohen

**Affiliations:** a Sharett Institute of Oncology, Hadassah Cancer Research Institute, Hadassah-Hebrew University Medical Center, Jerusalem, Israel; b Department of Dermatology, Hadassah-Hebrew University Hospital, Jerusalem, Israel; c The Wohl Institute for Translational Medicine, Hadassah-Hebrew University Medical Center, Jerusalem, Israel; d Department of Pathology, Hadassah-Hebrew University Medical Center, Jerusalem, Israel; e Department of Otolaryngology/Head & Neck Surgery, Hadassah-Hebrew University Medical Center, Jerusalem, Israel; f Department of General Surgery, Hadassah-Hebrew University Medical Center, Jerusalem, Israel

**Keywords:** cancer outcomes, COVID-19 pandemic, elderly patients, health care access, health care disruption, immunosuppression, medical surveillance, squamous cell carcinoma

*To the Editor:* The COVID-19 pandemic disrupted health care services, leading to delays in diagnoses and treatments, which may have exacerbated conditions like cutaneous squamous cell carcinoma (cSCC).^1^ While cSCC often has a favorable prognosis when diagnosed and treated promptly, delays in care may allow tumors to progress, increasing the risk of local invasion, recurrence, and metastasis.^2^ Elderly and immunosuppressed patients are generally considered the most vulnerable to such delays. This study evaluated whether COVID-19–related disruptions increased cSCC severity, with particular attention to these high-risk groups.

We conducted a single-center retrospective cohort study at Hadassah Medical Center, including patients aged ≥18 with biopsy-proven cSCC during 3 periods: pre-COVID-19 (1.6.2018-31.5.2019), during imposed restrictions (1.6.2020-31.5.2021), and postpandemic (1.6.2022-31.5.2023). Tumor severity was classified according to NCCN guidelines as nonsevere or severe (high or very high risk, based on size, histologic differentiation, perineural invasion, or high-risk anatomical location). Data extracted from electronic records included demographics, tumor features, recurrence status, and immunosuppression history. Group comparisons used Pearson chi-square tests (*P* < .05).

Among 511 patients (137 pre-, 188 during, and 176 postpandemic), mean age was 74.8 years. A nonsignificant increase in severe tumors was seen in the general population: 35.8% of cases pre-COVID-19, 47.8% during restrictions, and 43.2% postpandemic (*P* = .093).

In nonimmunosuppressed patients, severe tumors increased significantly during COVID-19 (36.5% to 58.1%, *P* = .022), with the sharpest rise among those ≥75 years (41.9% to 71.4%, *P* = .024). Conversely, in immunosuppressed patients, severity remained stable (*P* = .589, Fig 1).

**Fig 1:**
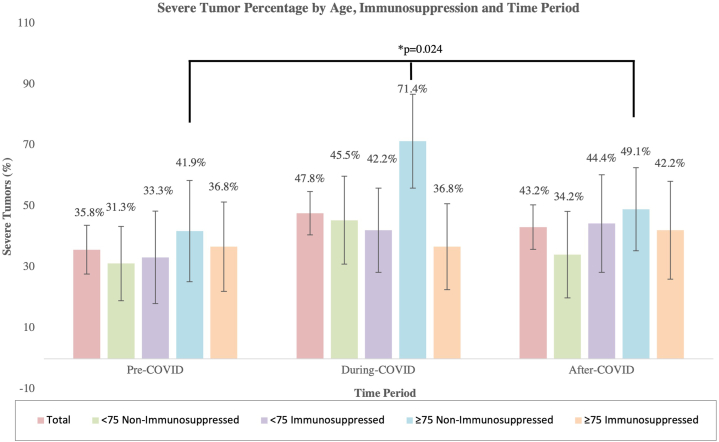
Percentage of severe cutaneous squamous cell carcinoma (cSCC) by age group (<75 y vs ≥ 75 y) and immunosuppression status across 3 time periods (pre-COVID-19, during COVID-19 restrictions, and post-COVID-19). Bars represent the proportion of severe tumors within each subgroup. Error bars indicate 95% confidence intervals.

The proportion of tumors in high-risk anatomical areas rose from 62.4% prepandemic to 72.8% postpandemic (linear association, *P* = .05, Fig 2). Contrary to expectations, severity did not differ between mask-covered and uncovered areas, indicating that pandemic-related diagnostic delays were more generalized rather than site-specific.

**Fig 2:**
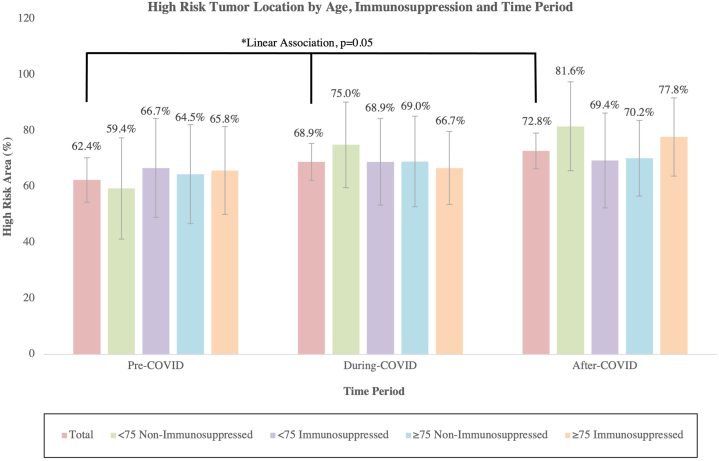
Percentage of cSCC located in high-risk anatomical sites (per NCCN guidelines) by age group (<75 years vs ≥ 75 years) and immunosuppression status across 3 time periods (pre-COVID-19, during COVID-19 restrictions, and post-COVID-19). Bars represent the proportion of tumors in high-risk sites within each subgroup. Error bars indicate 95% confidence intervals.

These findings underscore that pandemic-related care disruptions disproportionately affected elderly nonimmunosuppressed patients in our cohort, while immunosuppressed individuals likely benefitted from maintained follow-up due to their prior high-risk identification. The increase in tumors in high-risk locations highlights the importance of continuous access to dermatologic assessment even during public health emergencies.

Mitigation strategies should prioritize both access and triage. When facing limitations, maintaining in-person evaluations for high-risk patients, supported by teledermatology for triage and follow-up, could reduce the impact of future disruptions. Teledermatology has demonstrated 75% to 80% diagnostic concordance for skin neoplasms,^3^ and its integration with validated artificial intelligence–based image analysis could further enhance early detection. However, robust clinical validation is essential before widespread AI deployment.

Another area warranting further investigation is the potential effect of COVID-19 vaccination on cSCC progression, especially in immunosuppressed patients. Altered immune responses post-vaccination may theoretically influence tumor behavior, with some studies suggesting that immune modulation can affect skin cancer outcomes.^4^^,^^5^

Sustained dermatologic surveillance for vulnerable populations—especially elderly individual, both nonimmunosuppressed and immunosuppressed—is essential during health care system disruptions. Leveraging telemedicine and emerging diagnostic technologies may help preserve timely detection and treatment of cSCC during future crises.

## Conflicts of interest

Dr Cohen reports speaker honoraria from AstraZeneca, Bristol Myers Squibb, MSD, MedisonPharma, Merck Serono and Roche Pharma; Travel support from Gilead Sciences, Inc, Medison Pharma and Roche Pharma. Drs Korn, Sheffer-Levi, Eitan-Wexler, Ben-Shushan, Avner, Hirshhorn, Bracha, Mali, Shereberk-Hasidim, Merims, Popovtzer, Molho-Pessach, and Lotem have no conflicts of interest to declare.
